# Prevalence and associated factors of malnutrition among school going adolescents of Dang district, Nepal

**DOI:** 10.3934/publichealth.2019.3.291

**Published:** 2019-08-21

**Authors:** Sigma Bhattarai, Chet Kant Bhusal

**Affiliations:** 1 Nursing Department, Universal College of Medical Science and Teaching Hospital, Tribhuvan University, Bhairahawa, Rupandehi Nepal; 2 Department of Community Medicine, Universal College of Medical Science and Teaching Hospital, Tribhuvan University, Bhairahawa, Rupandehi Nepal

**Keywords:** adolescents, malnutrition, underweight, overweight, obesity, Dang

## Abstract

**Background:**

Malnutrition is a quiet emergency and one of the most widespread causes of morbidity and mortality among children and adolescent throughout the world; however there are very limited indications about the cause of malnutrition among adolescents. This study aimed to find out the prevalence and associated factors of malnutrition among school going adolescents of Dang district, Nepal.

**Methods:**

School based descriptive cross-sectional research design among 510 school adolescents studying in grade 9 and 10 between ages 14–17 years on April–October 2017 was conducted in Dang district Nepal. Among total 130 secondary schools, 10 schools were selected; one government and one private from each 5 electoral constituency using multistage probability random sampling.

**Results:**

The mean age and family size was 15.28 ± 0.77 and 5.25 ± 1.56 respectively. Among the total 25.7% of the adolescents are malnourished where 21.8% underweight, 3.1% overweight and 0.8% obese. After adjustment some of the variables such as religion (OR = 0.19; CI = 0.05–0.65, *p* = 0.008), family type (OR = 0.28; CI = 0.13–0.61, *p* = 0.001), school type (OR = 0.46; CI = 0.22–0.98, *p* = 0.044), earning status of family (OR = 4.52; CI = 1.44–14.16, *p* = 0.010), daily intake of green leafy vegetables (OR = 0.49; CI = 0.26–0.93, *p* = 0.031) and school sports (OR = 0.49; CI = 0.25–0.96, *p* = 0.040) were significantly associated with the underweight of adolescent. Similarly, variables such as gender (OR = 0.20; CI = 0.04–0.97, *p* = 0.046) and religion (OR = 9.75; CI = 2.24–42.39, *p* = 0.002) were significantly associated with the overweight/obesity of adolescent.

**Conclusion:**

Malnutrition was significantly higher among adolescents living in joint family, family having no earning status. Male adolescents were found more likely to be overweight and obesity. Hence to tie up the good nutrition it is recommended that integrated nutritional intervention and health related services should also be focused on adolescents.

## 1. Introduction

Malnutrition is a quiet emergency and it is one of the most widespread causes of morbidity and mortality among children and adolescents throughout the world [1],[2]. Malnutrition is a major public health problem throughout the developing world particularly in Southern Asia and Sub Saharan Africa [3]–[5]. In many of the developing countries, stunting, underweight, and micronutrient deficiencies among adolescents frequently result from inadequate nutrition and infections during early childhood combined with a diet insufficient to meet the intense nutritional demands of rapid growth during adolescence [6]. Adolescents are the persons of having age group 10–19 years. Adolescents experience a critical transition from childhood to adulthood, which is characterized by a rapid physical growth; psychological development and social changes [7]. Adolescents are nutritionally vulnerable due to their high requirements for growth, eating patterns and their susceptibility to environmental influences. Inadequate nutrition in adolescence can potentially retard growth and sexual maturation. Inadequate nutrition also puts adolescent at high risk of chronic disease although the detrimental effects appear after a long time [8]. Approximately 20% of the population of the WHO South-East-Asia (SEAR) consists of adolescents. The foundation of adequate growth and development is laid before birth, during childhood, and is followed during adolescence [9]. Adolescents in Nepal cover 23.45 percent of the total population that is nearly a quarter of population whereas they cover 22.34 percent of total population in Dang district [10]. Indicators of over nutrition such as overweight and obesity in children and adolescents now occur simultaneously with underweight, stunting and wasting [11]–[14]. The inconsistency of these two boundaries, repeatedly referred to as the “double burden of malnutrition” [14]–[16]. Obesity is a worldwide problem that is rising at an amazing rate. The children and adolescent obesity are the burning issues [17]. The World Health Organization reflects that poor nutrition is the single most significant threat to the world's health [18]. Poorer nutritional status becomes observable during adolescence, with a hindrance in maturation which may have consequence effect for consequent ability of the biologically immature female to bear a normal pregnancy [19]. The degree of the malnutrition is very high in Nepal [20]. According to Global School Based Student Health Survey (GSHS) 2015, 10.9% (male 13.8%, female 8.1%) adolescent students were underweight, 6.7% (male 7.6%, female 5.8%) were overweight (heavy for their height) and 0.6% (male 0.8% and female 0.4%) of the school going adolescent were obese [21]. A cross-sectional study conducted among school going adolescent girls, 9–16 years studying in various schools in rural area of Kavre district, Nepal found that overall prevalence of underweight, stunting and thinness was 31.98%, 21.08% and 14.94% respectively [22]. In most developing countries, nutrition initiatives have been focusing on children and women, thus neglecting adolescents. Addressing the nutrition needs of adolescents could be an important step towards breaking the vicious cycle of intergenerational malnutrition, chronic diseases and poverty [9]. Most of the studies are concerned with the nutritional status of under-five children and mother. Very limited research has been conducted to investigate the reason of having malnutrition among Adolescents in Nepal. Hence this study aimed to find out the malnutrition status and associated factors among school going adolescents of Dang district Nepal.

## 2. Materials and methods

### 2.1. Study design and source of population

Institutional based descriptive cross-sectional study was conducted in Dang district Nepal among secondary level school going adolescents studying in grade 9 and 10 between ages 14–17 years between July-December 2017. School adolescents both from government and private schools studying in grade 9 and 10 and between age group of 14–17 years of selected schools were included in the study while adolescents with severe mental problem and who were not available during the day of data collection were excluded in the study.

### 2.2. Sample size determination and sampling technique

**Figure 1.**
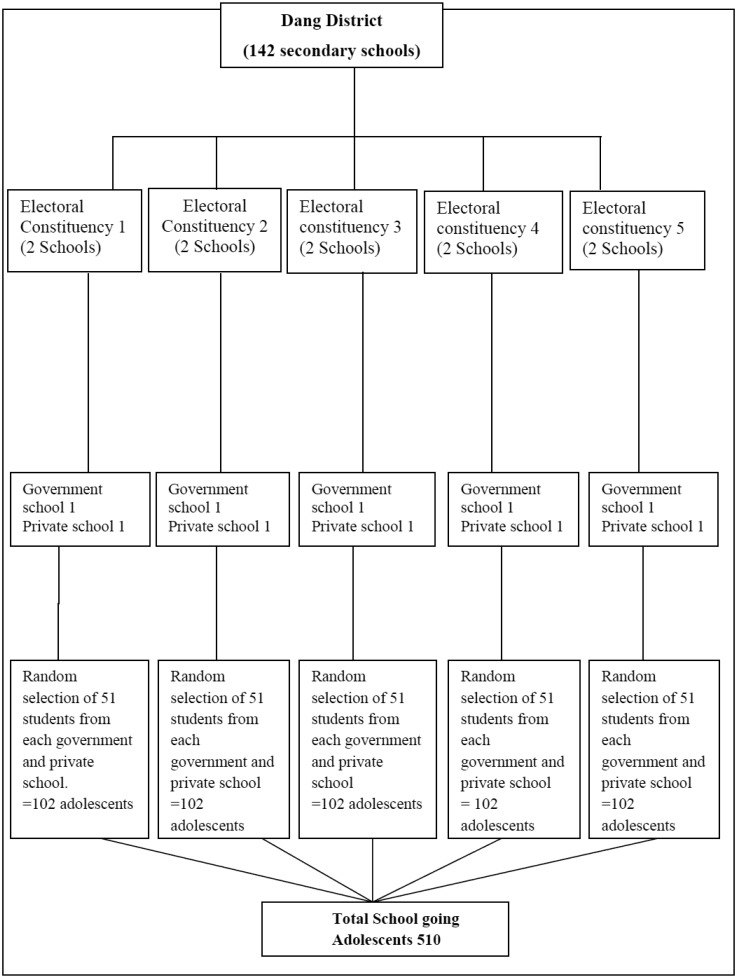
Multistage stratified probability random sampling technique.

The sample size of the study was 510 which was determined by using formula N = Z^2^pq/L^2^ [23] with 95% level of confidence interval, critical value Z=1.96, 3.5% margin of error, 10% non-response rate and 8.1% adolescents age 15–19 years from Kaski district were overweight and obese [24]. Hence, N = Z^2^pq/L^2^ = (1.96)^2^ × (0.081) × (0.919)/(0.035)^2^ = 232. Since multistage stratified probability random sampling was used as a sampling technique, initial sample size was multiply by design effect 2.0, hence n = 232 × 2 = 464. Further by adding 10% non-response rates i.e. 46, the final sample size of the study is 510. A multistage probability random sampling among total 142 secondary schools consisting of both government and private schools of Dang district was used. From each 5 existing electoral constituency, one government and one private school were selected by disproportionate stratified random sampling technique through non replacement lottery method. Further, 510 students was selected randomly from selected 10 secondary schools which consists 51 students from each government and private school by using non replacement lottery method (Figure 1).

### 2.3. Data collection procedures and validity

Pretested semi structure self-administered questionnaire was performed for collecting data. Questionnaire was translated into Nepali and then retranslated into English language to find misinterpretation and then correction was made. The self administered questionnaire was pretested among 10% of total sample size (51 school adolescents) residing in Birgunj, Parsa. Both English and Nepali version Questionnaire was made and used according to familiarity of students. Immediately after self-administered questionnaire anthropometric measurement was done. Height was measured in centimeter from head to heel by removing shoes with a non-stretchable measuring tape on the wall of school. In order to maintain accuracy and consistency of measuring tape, the same measuring tape was used to measure height throughout data collection period. The measuring tape was calibrated on a regular basis in the field against standard height measurement instrument. Weight was measured in kilogram with same standard calibrated digital weighing machine with the school adolescents standing with shoes removed and on school dress. Two data collectors including 1^st^ and 2^nd^ authors with qualification of Master in Nursing and Master in Public Health as well as Master in Sociology were involved in data collection.

### 2.4. Data processing and analysis

After data collection, data were thoroughly screened, reviewed, compiled and checked for its completeness, consistency and accuracy by the researcher and data analysis was done as per the objectives of the study. Editing, classifying, coding and entry of data were done using Microsoft Excel and analysis was done using Statistical Package for Social Science (IBM SPSS) version 20. Descriptive analysis such as frequencies, percentage, means and standard deviations were calculated. Bivariate and multivariate analysis was done to find out association between dependent and independent variables. Odds ratio and corresponding 95% confidence interval was used to find out the significance of association. Variables which were found statistically significant at 95% CI (*p* < 0.05) during bivariate analysis were further analyzed using logistic regression model in multivariate analysis (stepwise backward likelihood ratio method). The data were summarized, adjusted odds ratios (AORs) were estimated and their corresponding value at 95% confidence intervals (95% CI) was computed.

### 2.5. Setting

Dang district is located in the inner Terai and mid hills of Rapti zone in the mid-western development region of Nepal. Salyan and Rolpa are adjacent in the North, India in the south, Kapilvastu, Argakhachi and Pyuthan in the east, Surkhet and Bankae in the west [25]. During study time, there were 4 municipalities, 5 electoral consistencies and 31 VDCs out of which 5 VDCs lies in hilly region. According to Central Beuro of Statistics 2011 total population of Dang district was 552,583 among which 291,524 (52.76%) were female and 261,051 (47.24%) were male. Annual population growth rate of Dang district was 1.78 [10]. There are altogether 142 secondary schools including 86 private and 56 Government schools, similarly there are total 24,622 students in secondary level in Dang district which includes 12, 905 female and 11, 717 students [26].

### 2.6. Anthropometric measurement of adolescents

Anthropometric measurement of adolescent was done by using the standard calibrated digital weighing machine and non-stretchable measuring tape for weight and height respectively. After calculation of body mass index (BMI) through CDC BMI Percentile calculator software four indicators underweight, normal weight, overweight and obesity was recorded. CDC BMI Percentile calculator provides BMI and the corresponding BMI-for-age percentile based on the CDC growth charts. This calculator provides sex-specific CDC BMI-for-age growth charts where 5th percentile, between 5th percentiles to less than 85th percentile, between 85th percentiles up to less than 95th percentile, above 95th percentile of the sex specific CDC BMI-for-age growth charts gives underweight, normal weight, overweight and obesity respectively. For accuracy and consistency of anthropometric measurements, the same measuring tape and weighing machine was used to measure height and weight respectively throughout data collection period. The weighing machine and the measuring tape were calibrated on a regular basis in the field against standard weighing machine and height measurement instrument.

### 2.7. Ethics approval and consent to participate

This study obtained ethical approval from Institutional Ethical Review Board of National Medical College which is institutional review board of Nepal Health Research Council. Concerned stakeholders were officially contacted with letters and permission was obtained at all levels. Verbal informed consent was taken from parents and written informed consent was obtained from school teachers since the students were below 18 years old. Every study adolescents were informed about confidentiality and privacy.

## 3. Results

The mean age and mean family size was 15.28 ± 0.77 and 5.25 ± 1.56 respectively. Among total 510 school going adolescents two third (66.5%) of them were of less than equal to 15 years. Slightly more than half (51.4%) of total adolescents were male. In addition 42.7% of total adolescents were Brahmin/Chhetri followed by janajati, dalit, madeshi and muslim. Nearly two-third (64.3%) was from nuclear family. About half of the adolescents were from Government and Private schools respectively. Almost 12% of respondents' mother was found still unable to read or write and 6.4% of husbands and 13.6% of their wives had their informal classes. Nearly two-third (64.5%) of respondents' mother was engaged in household activities whereas about 45% of their fathers were engaged in different kind of services. Most 90.6% of the adolescents were from the families who have their own agricultural land (Table 1).

**Table 1.** Distribution of background related characteristics of study population.

General Characteristics	Frequency (n = 510)	Percentage
**Age**		
≤ 15 years	339	66.5
> 15 years	171	33.6
***Mean age ± SD; 15.28 ± 0.77***		
**Sex**		
Male	262	51.4
Female	248	48.6
**Ethnicity**		
Dalit	73	14.3
Janajati	150	29.4
Madeshi	3	0.6
Muslim	3	0.6
Brahman/chhetri	218	42.7
Others*	63	12.4
**Family Type**		
Nuclear	328	64.3
Joint	182	35.7
**Type of School**		
Government	257	50.4
Private	253	49.6
**Education of Mother**		
Illiterate	63	12.4
Informal education or primary (1 to 5)	172	33.7
Lower secondary level (6 to 8 class)	144	28.2
Secondary level (9 to 10 class)	64	12.5
SLC Passed	50	9.8
Intermediate +Bachelor and above	17	3.3
**Occupation of Mother**		
Homemaker	329	64.5
Small scale business	29	5.7
Service	36	7.1
Daily wage labor	11	2.2
Agriculture	105	20.6
**Occupation of Father**		
Small scale business	62	12.2
Service	229	44.9
Daily wage labor	40	7.8
Agriculture	121	23.7
Foreign job	58	11.4
**Own Agricultural Land**		12.4
Yes	462	90.6

Based on 24 hours dietary recalls methods, most of the participants (99%) consumed cereals, slightly more than three-fourth (76.9%) green vegetables and fruits, nearly one-third (65.7%) pulses and few 11.6% consumed meat/fish /eggs within 24 hours of data collection time. Regarding the school Tiffin of adolescents less than one-fifth (19.2%) carry homemade foods such as roti/paratha and vegetables along with them where as more than half (53.5%) consumed junk food such as noodles/lays during school break (Table 2).

**Table 2.** Distribution of Food Diversity related characteristics of study population within 24 hours time period.

Variable	Yes (%)	No (%)
Twenty four hours intake of cereals	508 (99%)	2 (0.4%)
Twenty four hours intake of Pulses	335 (65.7%)	175 (34.3%)
Twenty four hours intake of Vegetables/fruits	392 (76.9%)	118 (23.1%)
Twenty four hours intake of Meat/fish/eggs	59 (11.6%)	451 (88.4%)
Twenty four hours intake of Milk/ milk product	225 (44.1%)	285 (55.9%)
Tiffin food items		
Roti/paratha and vegetables	98 (19.2%)	412 (80.8%)
Packed food(noodles/lays)	273 (53.5%)	237 (46.5)
Fast Food from school canteen	139 (27.3%)	371 (72.7%)

The result about frequency of intake of food items showed that more than half 294 (57.6%) took junk food daily, nearly half 49.4% took three times meal in 24 hours. Nearly one-fourth (24.7%) of the respondents were vegetarians, among 384 non vegetarian adolescents, more than one-fourth (26.3%) adolescents took meat and fish occasionally, 35.6% took eggs occasionally. Among total, less than half (46.3%) took milk and milk product and 49.8% green leafy vegetables daily whereas less than one-fifth (17.8%) have habits of taking fruits daily in their diet (Table 3).

Regarding the nutritional status of the adolescents, among 510 adolescents almost three fourth (74.3%) had normal weight. Similarly 21.8% were underweight followed by overweight and obesity that is 3.1% and 0.8% respectively (Table 4).

Table 5 represents the multiple regression analysis of underweight with associated factors. Regarding religion the odds of underweight was found 0.19 times less likely (OR = 0.19, CI: 0.05–0.65) on Hindus rather than Christian. Similarly, adolescent living in Nuclear family was 0.28 times less likely (OR = 0.28, CI: 0.13–0.61) to have underweight than in joint family. Further, adolescents studying in Government schools were 0.46 times less likely (OR = 0.46, CI: 0.22–0.98) to be underweight than adolescents studying in private school. About the earning status adolescents whose family members were unable to earn to live their livelihood were more likely to have underweight by factor 4.52 (OR = 4.52, CI: 1.44–14.16) than the adolescents' whose family members earned money for their livelihood. In addition adolescents having daily intake of green leafy vegetables were significantly associated by factor 0.49 (OR = 0.49, CI: 0.26–0.93) than adolescent who did not take vegetables. Likewise, the adolescent who were not engaged in competitive sports were found 0.49 times less likely to be underweight (OR = 0.49, CI: 0.25–0.96) (Table 5).

**Table 3.** Distribution of frequency of intake of food items among study population.

Characteristics	Frequency (N = 510)	Percentage
**Frequency of having junk food**		
Daily	294	57.6
Twice a week	102	20.0
Weekly	35	6.9
Occasionally	79	15.5
**Frequency of meal in 24 hours**		
Two times	24	4.7
Three times	252	49.4
Four times	160	31.4
More than four times	74	14.5
**Vegetarian/Non vegetarian**		
Vegetarian	126	24.7
Non vegetarian	384	75.3
**Frequency of having meat/fish (n=384)**		
Daily	53	10.4
Twice a week	85	16.7
Weekly	110	21.6
Occasionally	134	26.3
**Frequency of having eggs (n=384)**		
Daily	33	6.5
Twice a week	77	15.1
Weekly	93	18.2
Occasionally	180	35.3
**Frequency of having milk and milk product**		
Daily	236	46.3
Twice a week	114	22.4
Weekly	45	8.8
Occasionally	115	22.5
**Frequency of having green leafy vegetables**		
Daily	254	49.8
Twice a week	256	50.2
**Frequency of having fruits**		
Daily	91	17.8
Twice a week	128	25.1
Weekly	34	6.7
Occasionally	257	50.4

**Table 4.** Prevalence of Nutritional Status of Adolescents.

Characteristics	Frequency (N = 510)	Percentage
Underweight	111	21.8
Normal Weight	379	74.3
Overweight	16	3.1
Obesity	4	0.8
Total	510	100

**Table 5.** Multiple regression analysis of underweight with associated factors.

Characteristics	Unadjusted OR (95% CI)	*P*-value	Adjusted OR (95% CI)	*P*-value
**Religion**				
Hindu	1		1	0.008*
Christian	3.85 (1.56–9.50)	0.002	**0.19 (0.05–0.65)**	
**Family Size**				
≤ 5 members	1		1	
> 5 members	3.21 (2.08–4.96)	< 0.001	0.78 (0.35–1.71)	0.538
**Family Type**				
Nuclear	1		1	0.001*
Joint	0.25 (0.16–0.39)	< 0.001	**0.28 (0.13–0.61)**	
**Type of School**				
Government	1		1	0.044*
Private	0.49 (0.32–0.76)	0.001	**0.46 (0.22–0.98)**	
**Education of Mother**				
Illiterate	1		1	
Informal or Primary	4.28 (1.67–10.99)	0.007	1.62 (0.47–5.57)	0.439
Lower Secondary	2.19 (0.92–5.20)		1.16 (0.41–3.26)	0.769
Secondary	2.06 (0.85–5.01)		1.55 (0.54–4.48)	0.410
SLC and Above	3.89 (1.51–10.01)	0.045	1.90 (0.65–5.55)	0.237
**Duration of food sufficiency**				
≤ 6 months	1		1	
> 6 months	2.24 (1.34–3.77)	0.002	1.54 (0.79–2.99)	0.197
**Earning status**				
Not earning	1		1	0.010*
Earning(cash)	9.43 (3.77–23.57)	< 0.001	**4.52 (1.44–14.16)**	
**History of 24 hours milk/milk product**				
No	1		1	
Yes	1.61 (1.04–2.49)	0.031	0.99 (0.52–1.87)	0.975
**Daily intake milk and milk product**				
Yes	1		1	
No	0.45 (0.29–0.70)	< 0.001	0.55 (0.29–1.05)	0.071
**Daily intake of green leafy vegetables**				
Yes	1		1	0.031*
No	0.591 (0.38–0.90)	0.015	**0.49 (0.26–0.93)**	
**Sleeping hours**				
≤ 6 hours	1		1	
> 6 hours	3.45 (1.63–7.33)	0.001	0.49 (0.16–1.50)	0.212
**School sports**				
No	1	0.002	1	0.040*
Yes	0.47 (0.24–0.77)		**0.49 (0.25–0.96)**	
**Sufferance from diseases**			1	
No	1	0.016		0.914
Yes	0.59 (0.38–0.90)		1.03 (0.57–1.84)	

*Significant (*p* < 0.05)—AOR in bold denotes significant

Table 6 presents the multiple regression analysis of overweight/obesity with associated factors. Overweight/obesity was found 0.20 times more likely in male adolescents than female adolescents (OR = 0.20, CI: 0.04–0.97). Likewise, overweight/obesity was found 9.75 times less likely in Hindus rather than Christian (OR = 9.75, CI: 2.24–42.39) (Table 6).

**Table 6.** Multiple regression analysis of overweight/obesity with associated factors.

Characteristics	Unadjusted OR (95% CI)	*P*-value	Adjusted OR (95% CI)	*P*-value	
**Gender**					
Male	1		1		
Female	3.96 (1.30–12.03)	0.007	**0.20 (0.04–0.97)**	0.046*	
**Religion**					
Hindu	1		1		
Christian	0.09 (0.03–0.29)	<0.001	**9.75 (2.24–42.39)**	0.002*	
**History of 24 hours milk/milk product**					
No	1		1		
Yes	0.32 (0.12–0.85)	0.017	5.82 (0.47–71.05)	0.167	
**Daily intake of eggs**					
Yes	1		1		
No	4.25 (1.27–14.19)	0.035	0.37 (0.09–1.48)	0.162	
**Daily intake of milk and milk product**					
Yes	1		1		
No	3.65 (1.30–10.20)	0.009	0.14 (0.01–1.22)	0.076	

*Significant (*p*<0.05)—AOR in bold denotes significant

## 4. Discussion

The present study revealed that 21.8% of school going adolescents between the age of 14–17 years in Dang district Nepal at the time of survey were underweight. The findings of the present study is similar to the study conducted by Roba KT, Abdo M and Wakayo T in Adama City, Central Ethiopia which found that 21.3% of adolescent girls were underweighted [27], this finding is also supported by Nepal Demographic Health Survey, 2011 where 25.8% of late adolescent girls cut off BMI value 18 [28]. However different studies such as study conducted in 2014 in Kaski district of Nepal, further analysis of Demographic and Health survey 2008 in Ghana 2015 documented the lower prevalence of underweight 15.3% and 13.8% respectively among adolescents [24],[29]. In spite of this, most of the studies conducted in rural areas of different countries such as study conducted in 2015 in Kavre district of Nepal among rural school going adolescent girls of 9–16 years, cross-sectional study conducted by Bisai, Bose, Ghosh and De, 2011 among rural school children aged 11–18 years of West Bengal, India and data from the Global School-based Student Health Survey (GSHS) in seven African countries between 2006 and 2010, by Taru, Hesham, David and Jason in 2014 from randomly selected schools going adolescents of 11–17 years showed the prevalence of underweight among adolescents were high 31.98%, 28.3% and 31.9% respectively than the present study [22],[30],[31].The variation of underweight in different study might be due to the difference in study setting of different schools, different age group of adolescents included in the study and methodologies applied in the study.

Regarding the prevalence of overweight and obesity the present study revealed that 3.9% of school going adolescents between the age of 14–17 years were found to be overweight and obesity. This finding is supported by the study conducted in Adama City, Central Ethiopia which found 4.3% of school adolescent girls were overweight and obesity [27]. However finding of this study is higher in compare to Nepal Demographic Health Survey, 2011 which reveals 2.9% overweight and obesity among late adolescent girls [32]. In spite the separate comparison of overweight and obesity, the present study revealed overweight and obesity 3.1% and 0.8% respectively among school going adolescents of 14–17 age groups. The finding of this study is slightly less in compare to study done by Acharya, Chauhan, Thapa, Kaphle and Malla in 2014 which revealed 5.8% overweight and 2.3% obesity among school adolescents [24]. Similar pattern was also seen in other studies such as study conducted in India by Vinoth, Shanthi , Lakshmi, Umakanthan and Fatima, 2016 in six schools in a semi urban area of Southern part of India revealed prevalence of more overweight and obesity as 10.9% and 6% respectively [33], next study conducted by Gurung and Gurung in 2014 among school going adolscents in Belgaum city in India documented the prevalence of overweight and obesity as 12% and 3.3% respectively [34] which are the higher than the present study. However, cross-sectional data from the Global School-based Student Health Survey (GSHS) conducted by Taru, Hesham, David, and Jason, 2014 in seven African countries indicate prevalence of obesity of Benin was 0.6% which is similar to the finding of the present study [31]. This disparity in over nutrition in different studies might be due to the different study setting, different age group of adolescents included in the study and methodologies applied in the study.

The current study showed statistical significant *p* = 0.001 between type of family and underweight. Consistently the study done by Pal, Pari, Sinha, Prakash and Dhara, 2016 in West Bengal state, India found significant association of underweight with family type, however showed different result of that adolescents of nuclear families (family size < 4) were more likely to be under nutrition [2] in contrast to this study thatadolescent living in Nuclear family was 0.28 times less likely to have underweight than in joint family. The finding of the present study found the significant association *p* = 0.010 of underweight with earning status of adolescents. Similar result was found in community based cross sectional study done by Pal, Pari, Sinha, Prakash and Dhara, 2016 in West Bengal India, which revealed poverty is found to be an important factor of under nutrition among the adolescents [2]. Adolescents' mother education in this study was found to be statistically significant (*p* = 0.007) with underweight in bivariate analysis. Similar pattern follows in most studies one of the study conducted by Vinoth, Shanthi, Lakshmi, Umakanthan and Fatima, 2016 in Southern part of India showed mother's education have significant associations with under nutrition [33]. Similarly a study conducted by Senbanjo, Oshikoya, Odusanya and Njokanma, 2011 in Abeokuta, Nigeria revealed that low maternal education was the major contributory factor to stunting [35]. Another cross sectional study done by Mukherjee, Chaturvedi and Bhalwar, 2008 found mothers' educational level was significantly associated with the nutritional status of the child [36]. Study done in West Bengal conducted by Pal, Pari, Sinha, Prakash and Dhara, 2016 showed adolescents of women with higher education were less likely to be undernourished than adolescents of poor and uneducated women [2].

The current study found gender of adolescent was significantly associated with overweight/obesity. Similar result was found in the study conducted by Goyal, Shah, Saboo, Phatak, Shah and Gohel, 2010 in school adolescent of 12–18 found age-adjusted prevalence of overweight was found to be 14.3% among boys and 9.2% among girls where as the prevalence of obesity was 2.9% in boys and 1.5% in girls [37]. However in contrast to the present study, Taru, Hesham, David and Jason, 2014 studied Cross-sectional study data from the Global School-based Student Health Survey (GSHS) in seven African countries among 11–17 years old school adolescents revealed that females had a higher overweight in five of the countries [31]. This contraction might be due to the male dominant Nepalese society.

The present study revealed that Christian was 0.19 times less likely to be underweight in comparison to Hindus. Similar result was found in the study conducted by Adeladza A. in 2009 in Kwale district of Kenya and Sabharwal NS in 2011 in rural India where Christian have better nutritional status [38],[39]. In concurrent with the present study nutritional status and religion was statistically significant in the study conducted by Arepalli S, Rao GV in Kallur Primary Health Center, Kurnool District of India in 2016 [40]. Similarly in this study overweight/obesity was found 9.75 times more likely in Christian rather than Hindus, this difference might be due to the difference in intake of diet, majority of Christian in the study area own cattle and engaged in agricultural work, however the finding of this study was supported by the study done by Peltzer et al., 2014in which high prevalence of organized religious activity was associated with overweight/obesity [41]. In simultaneous with the present study religious affiliation was associated with overweight/obesity in study conducted by Bharmal NH, McCarthy WJ, Gadgil MD, Kandula NR, Kanaya AM in 2018 [42].

## 5. Conclusion

The prevalence of underweight, overweight and obesity among school going adolescents between the age of 14–17 years in Dang district Nepal at the time of survey were 21.8%, 3.1% and 0.8% respectively. Prevalence of both under nutrition and over nutrition in school going adolescents demonstrated the existence of double burden of malnutrition. Malnutrition was significantly higher among adolescents living in joint family, family having no earning status. Male adolescents were found more likely to be overweight and obesity than female adolescents. Hence to tie up the good nutrition it is recommended that integrated nutritional intervention and health related services should also be focused on adolescents.
